# The Immune Cell Composition in Barrett's Metaplastic Tissue Resembles That in Normal Duodenal Tissue

**DOI:** 10.1371/journal.pone.0033899

**Published:** 2012-04-03

**Authors:** Alexandra Lind, Peter D. Siersema, Johannes G. Kusters, Jan A. M. Van der Linden, Edward F. Knol, Leo Koenderman

**Affiliations:** 1 Department of Respiratory Medicine, University Medical Center Utrecht, Utrecht, The Netherlands; 2 Department of Gastroenterology and Hepatology, University Medical Center Utrecht, Utrecht, The Netherlands; 3 Department of Medical Microbiology, University Medical Center Utrecht, Utrecht, The Netherlands; 4 Department of Dermatology, University Medical Center Utrecht, Utrecht, The Netherlands; Charité-University Medicine Berlin, Germany

## Abstract

**Background and Objective:**

Barrett's esophagus (BE) is characterized by the transition of squamous epithelium into columnar epithelium with intestinal metaplasia. The increased number and types of immune cells in BE have been indicated to be due to a Th2-type inflammatory process. We tested the alternative hypothesis that the abundance of T-cells in BE is caused by a homing mechanism that is found in the duodenum.

**Patients and Methods:**

Biopsies from BE and duodenal tissue from 30 BE patients and duodenal tissue from 18 controls were characterized by immmunohistochemistry for the presence of T-cells and eosinophils(eos). *Ex vivo* expanded T-cells were further phenotyped by multicolor analysis using flowcytometry.

**Results:**

The high percentage of CD4^+^-T cells (69±3% (mean±SEM/n = 17, by flowcytometry)), measured by flowcytometry and immunohistochemistry, and the presence of non-activated eosinophils found in BE by immunohistochemical staining, were not different from that found in duodenal tissue. Expanded lymphocytes from these tissues had a similar phenotype, characterized by a comparable but low percentage of αE(CD103) positive CD4^+^cells (44±5% in BE, 43±4% in duodenum of BE and 34±7% in duodenum of controls) and a similar percentage of granzyme-B^+^CD8^+^ cells(44±5% in BE, 33±6% in duodenum of BE and 36±7% in duodenum of controls). In addition, a similar percentage of α4β7^+^ T-lymphocytes (63±5% in BE, 58±5% in duodenum of BE and 62±8% in duodenum of controls) was found. Finally, mRNA expression of the ligand for α4β7, MAdCAM-1, was also similar in BE and duodenal tissue. No evidence for a Th2-response was found as almost no IL-4^+^-T-cells were seen.

**Conclusion:**

The immune cell composition (lymphocytes and eosinophils) and expression of intestinal adhesion molecule MAdCAM-1 is similar in BE and duodenum. This supports the hypothesis that homing of lymphocytes to BE tissue is mainly caused by intestinal homing signals rather than to an active inflammatory response.

## Introduction

Barrett's esophagus (BE) is a risk factor for the development of esophageal adenocarcinoma (EAC) with an incidence rate of around 1 in 200 patient years of follow-up in BE [1]. The incidence EAC continues to increase and is currently the fastest rising malignancy in the Western world [2].

BE is characterized by the presence of columnar epithelium of the intestinal type, which is mostly induced by gastroesophageal reflux [3]. The transformation of the normally present squamous lining in the esophagus into the intestinal-type columnar lining in BE is accompanied by the presence of high numbers of immune cells [2], [4]–[7]. This increase in immune cells is also observed in reflux esophagitis (RE), which most likely precedes the development of BE [2], [4], [8]. Currently, not much is known about the distribution of immune cells in RE in relation to the induction of BE. The presence of a chronic inflammatory reaction has, however, been associated with an increased risk of developing BE and progression towards neoplastic changes in this premalignant disorder [9], [10].

While no detailed studies have been performed on the distribution of immune cells in BE, earlier studies have suggested that the presence of T-cells seen in BE tissue is indicative of a Th2- response [7], [11]. Fitzgerald *et al* showed an increased expression of IL-4 mRNA in BE-tissue, which was four-fold higher compared to RE [11]. They also found indications for a Th1 response in esophageal tissue of RE as suggested by an upregulation of IFN-γ mRNA compared to BE (3–10-fold increase). These data were supported by immunohistochemical evidence showing enhanced staining for IL-4 and IFN-γ in frozen BE and RE sections, respectively [11]. In this study, esophageal metaplastic (intestinal type) tissue was compared with esophageal squamous epithelium of RE patients and controls.

Until now, BE has not been compared with another type of columnar epithelium, such as duodenum. This may be relevant as even in the absence of an ongoing inflammatory response the normal gut tissue is relatively rich in Th2 type T-lymphocytes [12]. These observations prompted us to investigate an alternative hypothesis, i.e., that immune cells in BE tissue are in fact present as a consequence of intestinal-type of columnar epithelium in BE rather than being a result of an active inflammatory response.

Previous studies on the immune cell composition in BE have mainly focused on PCR results of whole biopsies or immunohistochemistry on BE sections due to the relatively small amount of biopsy material that can be obtained from patients [7], [11], [13]. The main drawback of immunohistochemistry is; however, that a simultaneous analysis of T-cells subsets or markers on these cells in a single slide is not possible. Recently, Clark *et al* reported a method which allowed immunophenotyping of T-cells cultured from small skin biopsies [14]. This technique uses a three-dimensional growth matrix (collagen-coated carbon matrix) on top of which a small skin biopsy is placed. Under these conditions, fibroblasts can grow into the matrix, while T-cells detach from the matrix and proliferate in the culture medium. T-cells were found to expand at least 10-fold and various T cell populations, particularly those that were positive for skin-homing chemokine receptors, were detected. This method provides an opportunity to amplify the numbers of various T-cell populations in biopsies from the esophagus and allowed multicolor analysis and functional experiments with T-cells.

In this study, we therefore performed *in vitro* expansion of T-cells from biopsies of BE and duodenal tissue from BE patients and duodenal tissue in order to allow us to analyse T-cell (sub) population in these tissues in more detail.

## Results

### Absence of activated eosinophils in BE and duodenum

**Figure 1:**
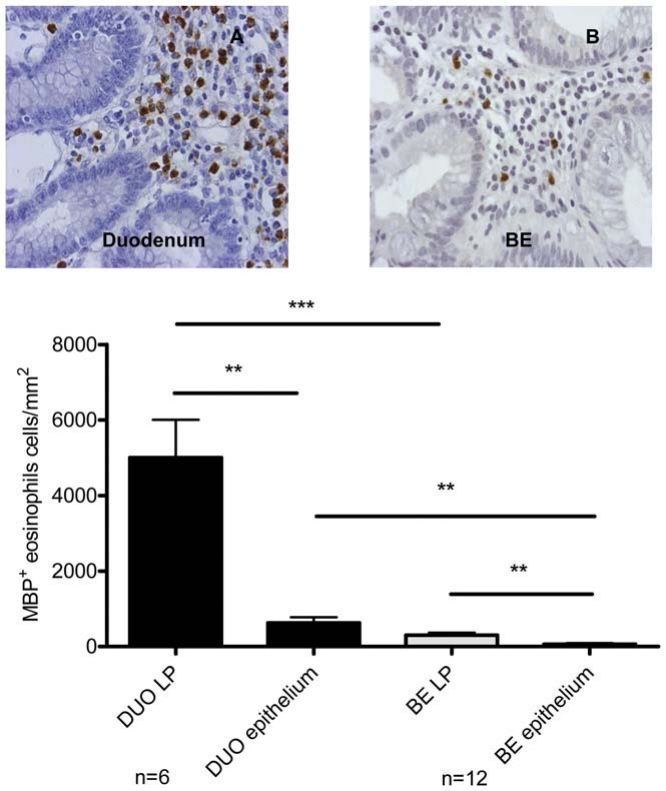
Similar morphology of eosinophils in Barrett's esophagus (BE) and duodenum. Immunohistochemical staining with anti-MBP (eosinophil marker) of duodenum from 6 controls (black bars, panel A) and BE biopsies from 12 BE patients (light grey bars, panel B). Eosinophils were counted in lamina propria and intestinal epithelium separately. DUO: duodenum, LP: lamina propria. Each bar represents mean values±SEM and expressed as MBP-positive cells/mm^2^ (** p<0.005, *** p<0.0005).

Immunohistochemical staining of the biopsies for MBP was used to determine the presence of eosinophils. Twelve BE and 6 duodenal biopsies were compared. The number of eosinophils in lamina propria (LP) and epithelium were determined separately. LP and epithelium of BE biopsies were characterised by the presence of non-activated eosinophils (normal round cells with absence of free granules), but contained significantly lower number of eosinophils compared to LP (p<0.0009) and epithelium (p<0.002) of duodenum (Figure 1.) Only in 1/12 BE biopsies, free eosinophil granules were observed.

### High numbers of CD4^+^- cells in the lamina propria of BE and duodenum

**Figure 2:**
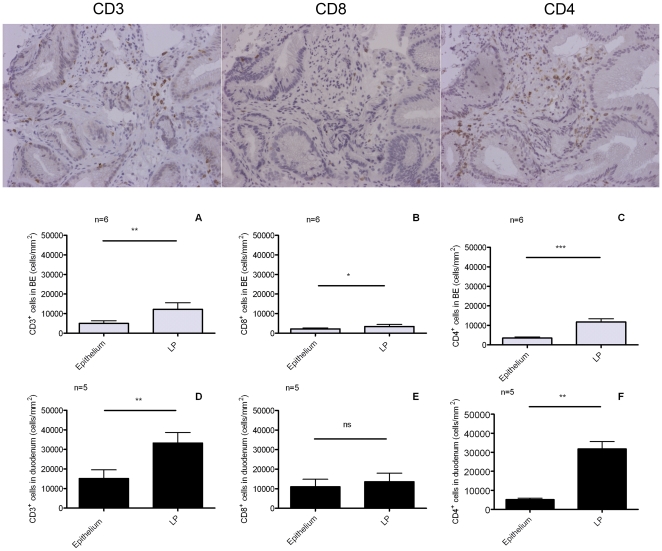
Immunohistochemical analysis of inflammatory cells in BE and duodenum. **Panels A–C Barrett's esophagus (BE).** Immunohistochemical staining for CD3^+^(panel A), CD8^+^(panel B) and CD4^+^-cells (panel C) was performed in BE biopsies from 6 BE patients. Cells were counted in lamina propria (LP) and epithelium separately (light grey bars, panels A and B). Each bar represents mean value±SEM and the numbers of CD3^+^, CD4^+^- or CD8^+^ are expressed as cells/mm^2^(n = 6). **Panels D–F Duodenum.** Immunohistochemical staining for CD3^+^(panel D), CD8^+^ (panel E) and CD4^+^-cells (panel F) was performed in duodenal biopsies from 5 controls. Cells were counted in lamina propria (LP) and epithelium separately (black bars, panels A and B).Each bar represents mean value±SEM, and the numbers of CD3^+^, CD4^+^- or CD8^+^-are expressed as cells/mm^2^ (*p<0.05, ** p<0.005, ***p<0.0005, ns not significant) (n = 5).

Immunohistochemical staining for CD3, CD4 and CD8 was performed to characterise lymphocytes in BE and duodenum of controls. In duodenum high numbers of CD3^+^ cells were found (33±5×10^3^ cells/mm^2^ in LP and 15±4×10^3^ cells/mm^2^ in epithelium). Significantly fewer CD3^+^ cells were found in BE (12±3×10^3^ cells/mm^2^ in LP (p<0.05) and 5±0.7×10^3^ cells/mm^2^ in epithelium (p = 0.05)) (Figure 2, panels A–C). CD8^+^-counts were low in LP of BE (3±1×10^3^ cells/mm^2^). The CD8^+^-count was relatively high in epithelium of BE (2±0.5×10^3^ cells/mm^2^) and in epithelium of duodenum (11±4×10^3^ cells/mm^2^) (Figure 2, panels B and E).

The interpretation of data regarding CD4^+^-cells in tissue is complex as part of the CD4^+^-cells are monocytes. Analysis of double stained (for CD3^+^CD4^+^-cells) paraffin embedded tissue did not result in interpretable results (not shown). As a result, the CD4^+^-T-cell count was estimated by subtracting the CD8^+^-cell count from the CD3^+^-cell count ( = total T-cells). The corrected CD3^+^CD4^+^ count in the duodenum was 5±2×10^3^ cells/mm^2^ in the epithelium and 20±2×10^3^ cells/mm^2^ in LP. The corrected CD3^+^CD4^+^-count in BE was 2±0.4×10^3^ cells/mm^2^ in epithelium and 6±2×10^3^ cells/mm^2^ in LP of BE (p<0.01).

### Comparable proportions of CD3^+^CD4^+^ T-cells in BE and duodenum from BE patients and controls

As immunophenotyping of tissue T-cells by immunohistochemistry only allows a limited characterization of these cells, we analysed these cells in more detail by FACS.

**Figure 3:**
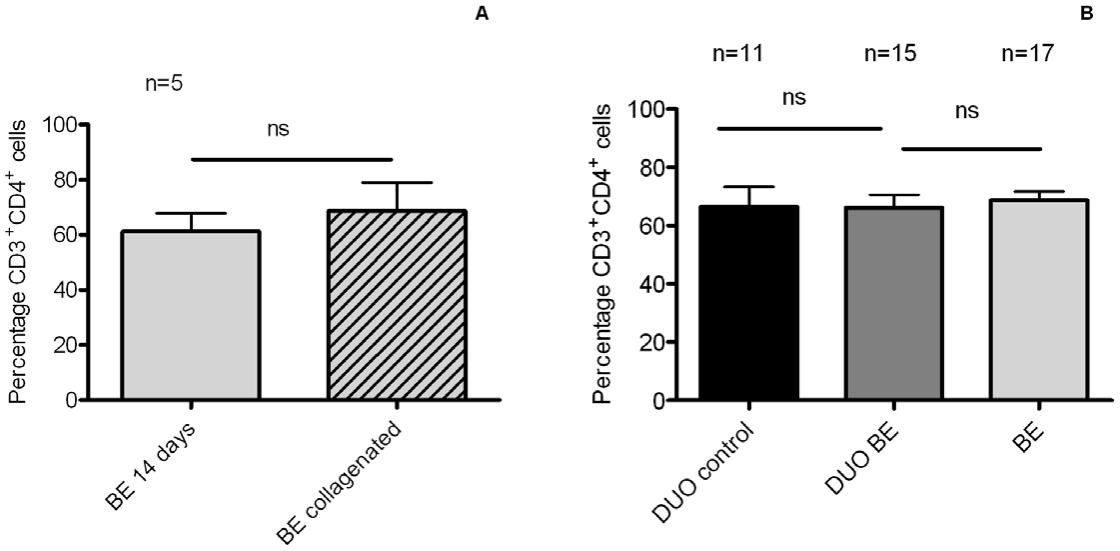
Similar percentage of CD3^+^CD4^+^-cells in BE and duodenal *ex vivo* cultures. (**A**) **Comparison of the percentage of CD3^+^CD4^+^-cells in collagenated BE biopsies and** ***ex vivo*** **expanded T-cells.** The percentage of CD3^+^CD4^+^-cells was determined in paired biopsies from 5 BE patients treated by either collagenase or used for *ex vivo* expansion. Each bar represents the mean value±SEM of the percentage of CD3^+^CD4^+^-cells in the CD3^+^population (light grey bar: BE T-cells expanded for 14 days *ex vivo*, light grey with stripes: T-cells from collagenated BE biopsies). (**B**) **Percentage of CD3^+^CD4^+^-cells in** ***ex vivo*** **cultures of BE and duodenum.** The percentage of CD3^+^CD4^+^-cells from *ex vivo* cultures of 17 BE segments and 15 duodenal biopsies from 17 BE patients (DUO BE) and 11 duodenal biopsies from 11 controls (DUO control) was determined by flowcytometry. Each bar represents the mean value±SEM of the percentage of CD3^+^CD4^+^-cells in the CD3^+^ population (black bar: DUO control, dark grey bar: DUO BE, light grey bar: BE) (**ns** not significant).

From five patients we obtained paired biopsies for collagenase treatment and *ex vivo* culture for 14 days. T-cells were directly isolated from the tissue after collagenase treatment of 10 biopsies and the phenotypes were compared with *ex vivo* expanded T-cells of the same patients by FACS. The mean percentage of CD3^+^CD4^+^-cells in collagenated BE biopsies was 64±8% (of all CD3^+^ cells), which was similar as found at day 14 of culture (p = 0.7) (Figure 3A). In one patient, sufficient material was obtained (biopsies for collagenase and T-cell culture) to follow the proportion of CD3^+^CD4^+^-cells at different time points. The proportion of CD3^+^CD4^+^-cells on day 0, 7 days and 14 days of culture was similar (73%, 65% and 72%, respectively).

Next, cells from BE and duodenum were evaluated after 2 weeks of culture. The proportion of CD3^+^CD4^+^ (of all CD3^+^ cells) in duodenum of controls (66±7%) and duodenum of BE patients (66±4%) was similar (Figure 3B). In addition, the proportion of CD3^+^CD4^+^-cells in cultures of biopsies from BE (69±3%) and duodenum from BE patients was also similar (Figure 3B). CD4^+^ and CD8^+^-cells from all *ex vivo* cultures were predominantly memory T-cells (CD45RO^+^) (99±2%). CD4^+^ cells from collagenated biopsies were found to be for 95% CD45RO^+^ and CD8^+^ cells for 85% CD45RO^+^.

### Similar proportions of CD4^+^CD103^+^- cells in BE and duodenum from BE patients and controls

**Figure 4:**
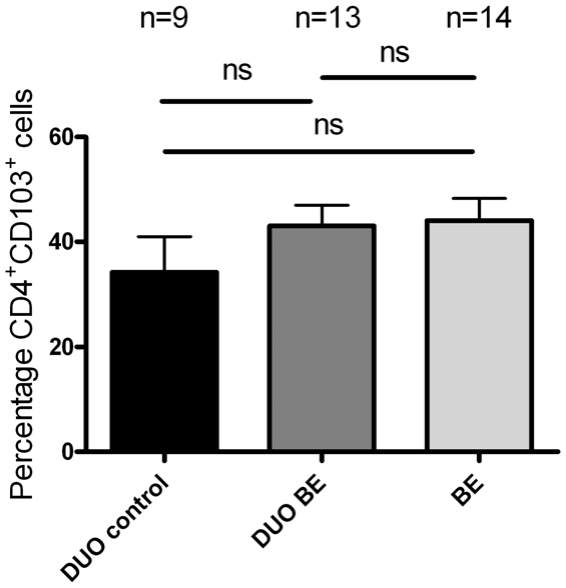
Low percentage of CD4^+^CD103^+^-cells in ex-vivo cultures of BE and duodenal tissue. The percentage of **CD4^+^CD103^+^-cells** in *ex vivo* cultures from 14 BE segments and 13 duodenal tissues from 14 BE patients (DUO BE) and 9 duodenal tissues from 9 controls (DUO control) was determined by flowcytometry (**ns** not significant). Each bar represents mean value±SEM, percentage of **CD4^+^CD103^+^-cells** in the **CD3^+^CD4^+^-cells** population, (black bar: DUO control, dark grey bar: DUO BE, light grey bar: BE).

CD103 (αE) is a subunit of the intraepithelial integrin αEβ7 and is present on T-cells with an immunosuppressive phenotype [15]. Duodenum of controls and BE had a similar proportion of CD4^+^CD103^+^ cells (34±7% and 43±4% of CD3^+^CD4^+^ cells, respectively) (Figure 4). *Ex vivo* cultures of BE had a similar proportion of CD4^+^CD103^+^ (44±4%) compared to cultures of duodenum from controls and BE (Figure 4).

### Similar proportions of CD8^+^Granzyme B^high^- cells in BE and duodenum from BE patients and controls

**Figure 5:**
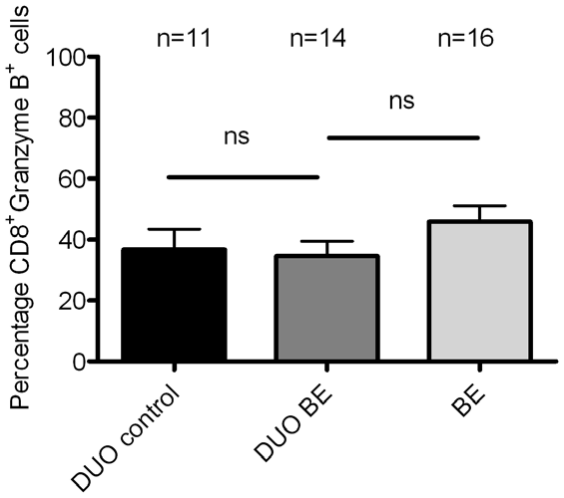
High percentage of CD8^+^Granzyme B^high^ cells in *ex vivo* cultures of BE and duodenal tissue. The percentage of Granzyme B**^high^** -cells (from CD8^+^ cells) was measured by an intracellular FACS-staining in *ex vivo* cultures of 16 BE segments and 14 duodenal tissues from 16 BE patients (DUO BE) and duodenum from 11 controls (DUO controls). Each bar represents mean value±SEM of the percentage **CD8^+^Granzyme B^high^ cells** in the CD3^+^CD8^+^ population (black bar: DUO control, dark grey bar: DUO BE, light grey bar: BE) (**ns** not significant).

To further phenotype CD8^+^-cells, intracellular granzyme B-staining in lymphocytes cultured from *ex vivo* biopsies was performed. The proportion of CD8^+^Granzyme B^high^-cells (of all CD8^+^-cells) in duodenum of controls was 37±6%, which was similar to duodenum of BE patients (35±5%) (Figure 5). The proportion of CD8^+^GranzymeB^high^ in *ex vivo* cultures of BE was 46±5%, which was not significantly different from *ex vivo* cultures of duodenum (Figure 5).

### Non-detectable IL-4 in T-cells from BE patients

**Figure 6:**
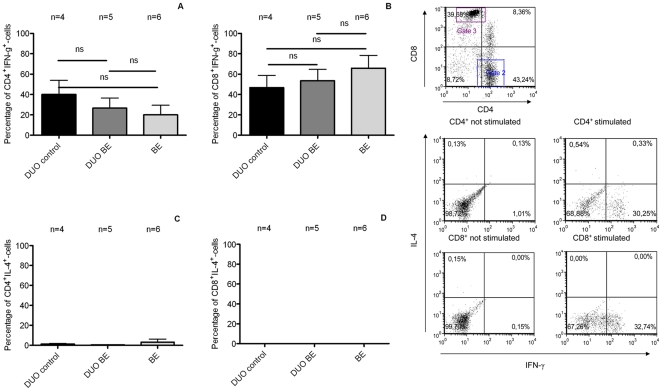
Absence of IL-4 positive lymphocytes in BE cultures determined by intracellular FACS-staining. (**1**) **Intracellular staining for IFN-γ and IL-4.** Intracellular staining for IFN-γ and IL-4 in T-cells was determined after stimulation with PMA (20 ng/ml) and ionomycin (1 µM) for 6 hrs. Hereafter, CD4 ^+^ and CD8^+^ cells from ex-vivo cultures of BE and duodenum from BE (DUO BE) and duodenum from controls (DUO control) were analysed. Each bar represents mean value±SEM of the percentage of IFN-γ cells inside CD3 ^+^CD4^+^-population (panel A), percentage of IFN-γ cells inside CD3^+^CD8^+^-population (panel B), percentage of IL-4 cells inside CD3^+^CD4^+^-population (panel C) and percentage of IL-4 cells inside CD3^+^CD8^+^-population (panel D) (black bar: DUO control, dark grey bar: DUO BE, light grey bar: BE) (**ns** not significant) (BE, n = 6,DUO BE, n = 5, DUO control, n = 4). (**2**) **Representative flowcytometry plots for IFN-γ and IL-4** Representative flowcytometry plots of a representative staining of lymphocytes from a duodenal culture *ex vivo*. Gates for CD4 and CD8 were set and positive cells for IL-4 and IFN-γ were determined.

Intracellular staining for IFN- γ and IL-4 was performed after 21 days of culture (to obtain sufficient number of cells) to evaluate the presence of effector Th2-cells in BE. The analysis was performed on T-cells from BE and duodenum from 6 BE patients and duodenum from 4 controls. T-cells from BE were characterised by a positive staining pattern for IFN-γ in CD4^+^ (20±9% cells) and CD8^+^-cells (66±13%) (Figure 6 A+B), which was similar to T-cells from duodenum of BE (CD4^+^-cells (27±10%) and CD8^+^-cells (54±11%))(Figure 6 A+B). There was no difference between duodenum of controls and BE in expression of IFN-γ on CD4^+^-cells (40±10% in duodenum of controls, p = 0.9 vs duodenum of BE) and CD8^+^-cells (47±12%, p = 0.6)(Figure 6 C+D). We were also not able to detect IL-4 in CD4^+^ and CD8^+^-cells in *ex vivo* cultures of duodenum of BE and controls, nor in *ex vivo* cultures of BE (Figure 6 C+D). We could clearly identify positive and negative populations in IL-4^+^ staining as T-cells from one ex vivo culture of one a BE patient had small population of IL-4^+^-cells.

### Similar expression patterns of α4 and β7 expressing integrins on T-cells in BE and duodenum of BE patients and controls

**Figure 7:**
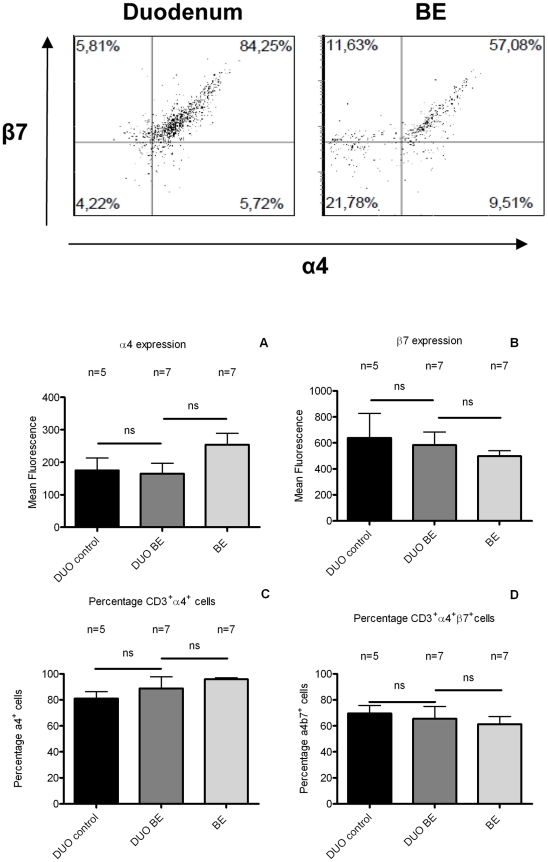
Comparable integrin expression on T-cells from BE and duodenal tissue. The expression was analysed of α4 (panel A) and β7 (panel B) (arbitrary units, mean fluorescence) by CD3^+^ cells in *ex vivo* cultures of BE and duodenum of BE patients (DUO BE) and controls (DUO control) (black bar: DUO control, dark grey bar: DUO BE, light grey bar: BE). Also, the percentages of CD3^+^α4^+^-cells (panel C) and CD3^+^α4β7^+^-T-cells (panel D) of the CD3^+^-population in ex-vivo cultures of BE and duodenum from both groups were determined (**ns** not significant). There was no difference in expression of α4 and β7 and percentage of α4^+^ and α4^+^β7^+^ cells between duodenum of BE patients (DUO BE, n = 7) and duodenum of controls (DUO control) (n = 5).

CD3^+^-cells from BE had a similar expression of the gut homing integrins α4 and β7 subunits (α4: 254±35; β7 498±42/mean fluorescence (MFI) ± SEM expressed in arbitrary units (AU)), which was not different from CD3^+^-cells from duodenum of BE (α4: 165±21 (AU), p = 0.1 vs. BE; β7: 583±100 (AU), p = 0.1 vs. BE) (Figure 7 A+B). There was also no difference in α4 and β7 expression on CD3^+^-cells between duodenum of BE and controls (Figure 7 A+B). The proportion of CD3^+^α4^+^-cells was similar in *ex vivo* cultures from BE (96±1%), duodenum of BE (89±9%) and duodenum of controls (95±3%) (Figure 7 C+D). The percentage of CD3^+^α4β7^+^-cells was also similar in BE (61±6%), duodenum of BE (65±9%) and duodenum of controls (69±6%) (Figure 7 C+D).

### MAdCAM-1mRNA expression in BE tissue is similar to MAdCAM-1 expression in duodenal tissue

**Figure 8:**
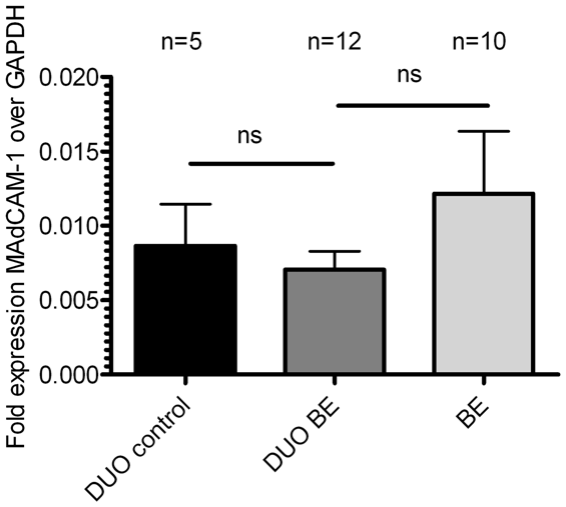
MAdCAM-1 mRNA expression is similar in BE and duodenal tissue from BE patients and controls. Real-time quantitative polymerase chain reaction RNA assays were performed on total BE biopsies (BE, n = 12), duodenal biopsies from BE patients (DUO BE, n = 10) and duodenal biopsies from controls (DUO controls, n = 5) (black bar: DUO control, dark grey bar: DUO BE, light grey bar: BE). Expression of MAdCAM-1, corrected for GAPDH, 2^−ΔCT^±SEM, is represented on the y-axis (ns: not significant).

MAdCAM-1 (mucosal vascular addressin cell adhesion molecule-1) is a ligand of α4β7 and is normally expressed on vascular endothelium of the intestinal lamina propria [12]. Expression of MAdCAM-1 in BE biopsies (0.01216±0.004200, 2^−ΔCT^±SEM, corrected for GAPDH) was similar to expression of MAdCAM-1 in duodenal biopsies from BE patients (0.007052±0.001244) and controls (0.008665±0.002790) (Figure 8).

## Discussion

This is the first study showing that the T-cell composition in tissue from BE (epithelium and lamina propria) is similar to what is found in duodenal tissue from BE and controls. As the specialized intestinal epithelium in BE esophagus is to a large extent similar to epithelium normally found in the duodenum, this suggests that the immune cell composition observed in BE is not an indication of an active Th2 type inflammation, as has been suggested before [7], [11], but rather a consequence of metaplastic intestinal-type changes that have occurred in BE.

The morphology of eosinophils in BE and duodenum [16], [17] exhibited a similar non-polarized, not-activated phenotype, which is in contrast to eosinophils found in allergic disorders that are polarized with the presence of free granules [18], [19]. This finding is the first indication that immune cells in BE are similar to those normally found in intestinal tissue and that their presence is likely not to be a consequence of inflammation. The observed slight difference in numbers of eosinophils in BE and duodenum may be explained by an aberrant intestinal microenvironment and/or a the known less well developed vasculature in BE [20], [21].

The high numbers of lymphocytes in BE were previously interpreted as evidence of an inflammatory response [7], [11]. However, little data are available regarding the specific phenotypes of these T-cells because of the limited analytic power of IHC to do this. Therefore, we applied a novel technique to expand *ex vivo* T-cells to analyze T-cell phenotypes by multicolor FACS analysis. Comparing this technique with IHC stainings showed similar numbers of CD3^+^/CD4^+^ and CD3^+^/CD8^+^-cells (Figures 2 and 3).

In our study, similarly high percentages of memory CD4^+^-cells were observed in *ex vivo* cultures of duodenum and BE. It is known that a healthy duodenum has abundant lymphocytes [22]. The homing of these cells to this tissue is mediated by a large repertoire of gut homing signals such as specific chemokines and chemokine receptors [23]. This coincides with the proximity of mesenteric lymph nodes and Peyer's patches [24]–[26]. The absolute numbers of lymphocytes in BE were lower than found in the duodenum. The underlying mechanism remains to be elucidated, but structural differences between BE and duodenal tissue might well be involved, for example, as suggested above, a more extensive vasculature in the duodenum compared to BE tissue that may facilitate lymphocyte homing to intestinal tissue [16], [20]. In addition, homing of T-cells from Peyer's patches towards the submucosa may increase the numbers of T-cells found in the duodenum [12], [23].

Despite the 2.5 fold difference in absolute numbers, the relative numbers of CD3^+^/CD4^+^ and CD3^+^/CD8^+^-cells in BE were similar as found in duodenum (Figure 2). Although the high number of CD3^+^/CD4^+^ does not rule out the presence of inflammation, these findings suggest that these immune cells are at least partly present in BE tissue due to homing signals normally found in the gut rather than inflammatory signals. In contrast to this view, previous studies have suggested that BE is associated with a Th2 inflammatory response based on the presence of mRNA for IL-4 and some IL-4 positive cells in IHC analysis [7], [11], [27]. In contrast, intracellular stainings for IFN-γ and IL-4 in our T-cell cultures did not show a significant expression of IL-4 in either CD4^+^ or CD8^+^-T-cells from BE cultures (Figure 6). In fact, a high percentage of IFN-γ-producing lymphocytes (both CD4 and CD8, see Figure 6) was found in both BE and duodenal tissue. Other studies have also reported the production of IFN-γ by lymphocytes obtained from healthy duodenum [28]. This is a further indication that the IFN-γ signal in BE is probably caused by homeostatic signals normally present in intestinal tissue rather than an inflammatory process.

Granzyme B^+^ CD8^+^-cells are effector cells in viral infections and cancer immunology [29], [30]. Our results showed similar proportions of Granzyme B^+^CD8^+^- cells in BE and in duodenal cultures from BE and controls (Figure 5) as has previously been reported for the duodenum; however, were lower than found in duodenal inflammatory disease [22]. These findings are further supportive of gut homing signals for T-cells in BE tissue (Figure 5).

The lymphocyte surface integrin CD103/beta7 (αEβ7) recognises epithelial cell E-cadherins involved in lymphocyte/epithelial cell interactions [31] and is associated with T-cells with an immunosuppressive phenotype [32]–[35]. Epithelial derived TGF-β controls the expression of this integrin [15]. The percentage of CD4^+^CD103^+^-cells was similar in BE and duodenum from BE patients and controls (Figure 5). The relative deficit of CD4^+^CD103^+^-cells in BE and intestine [36] may well reflect a gut homing mechanism of these cells. Interestingly, BE has been reported to be low in TGF-β and SMAD4 expression, particularly when compared with normal squamous epithelium [37]. This might well explain the low proportions of CD4^+^/CD103^+^-T-cells. In contrast, healthy duodenal tissue is associated with a high expression of TGF-β [38]. As this latter study did not compare TGF-β expression between BE tissue and healthy intestinal-like tissue no solid conclusions can be drawn with regard to TGF-β function in BE.

Finally, our hypothesis is supported by the expression of the intestinal homing integrin α4β7 on T-cells in BE which was similar to that found on the intestinal T-cells from the duodenum (Figure 7). Expression of the ligand for α4β7, MAdCAM-1 was also similar in BE tissue and duodenal tissue. This provides the conditions for lymphocyte arrest and trans-endothelial migration to BE tissue by a mechanism normally operational during homeostatic homing to intestinal tissue (Figure 8) [12]. The question then arises what the consequences are of the presence of “intestinal” lymphocytes in BE tissue. This is currently unknown as it is also unclear why intestinal-type columnar epithelium of the esophagus is associated with an increased risk of adenocarcinoma, while the malignant potential of duodenal tissue is low to almost not existent. As we hypothesize that the immunologic environment in BE is similar to that found in duodenal tissue, we speculate that the microenvironment (reflux of gastric contents) in Barrett's esophagus plays a causative role in the increased risk of neoplastic behaviour of this tissue.

This study is limited by the small size of the available biopsy samples taken during routine endoscopic examinations of our patients. Direct isolation of lymphocytes from these biopsies by collagenase treatment resulted in low numbers of cells, which were only sufficient for a single cell surface staining (intracellular staining was not possible). In order to allow a more detailed phenotypic characterization of the T-cells the immune cells present in these biopsy samples were expanded by in vitro culture. The main concern in the interpretation of the analysis of the *ex vivo* expanded cell populations is in a putative skewing of the T-cells during 14 days of culture. This method of *ex vivo* T-cell expansion has, however, been extensively validated for T-cells from skin tissues in which it was shown that both the chemokine receptors and homing properties of the cells remained intact [14]. In this study, we validated this method for BE by comparing the phenotype of *ex vivo* expanded T-cells with that of cells isolated directly from the BE biopsy by collagenase treatment. The proportion of CD3^+^/CD4^+^ cells after 14 days of culture of BE biopsies was similar to that found in collagenated BE biopsies (Figure 3A). Moreover, the immunohistochemical analysis of stained tissue sections confirmed the predominant presence of CD3^+^/CD4^+^ cells in BE and duodenal tissue. A drawback of the tissue staining was that CD4^+^ cells comprise more than T-cells. We therefore calculated the number of CD4^+^ T-cells by subtracting the number of CD8^+^ T-cells from the CD3^+^ count. CD3^+^ cells can also include natural-killer cells, but it is accepted to disregard them, as they have been shown to comprise only 0.34% of the total CD3^+^ population in the duodenum [39]. Also, no CD3^+^CD16^+^ cells were seen in *ex vivo* cultures and collagenated biopsies from duodenum and BE.

In conclusion, the composition of immune cells in BE tissue was very similar to that found in normal, non-inflamed duodenal tissue. In addition, the lymphocytes and eosinophils observed in BE tissue were also not different in characteristics compared to those in normal non-inflamed duodenal tissue. The expression of the adhesion molecule MAdCAM-1 both in BE and duodenal tissues supports the hypothesis that the inflammatory cell composition in BE is not characterized by an active inflammatory process, but rather by a change in the immune composition driven by an altered homing due to the metaplastic changes in BE. This suggests that the influx of inflammatory cells during the metaplasia to BE is not mainly caused by an inflammatory process but in fact is the consequence of alterations due to the metaplastic tissue more resembling duodenal tissue. Further studies on the role of these “intestinal” lymphocytes in BE may result in a better understanding of the pathogenesis and/or prognosis of BE.

## Materials and Methods

### Patient characteristics

Fifty-nine patients were included in our study. Of these, 41 patients had BE, as defined by the presence of specialised intestinal metaplasia (IM) containing goblet cells in at least one of the biopsies. Eleven patients were excluded from the BE group due to the presence of macroscopic esophagitis (ulcers and erosions) proximal to the Barrett's segment (n = 1), BE segments being smaller then C0M2 because of the risk of biopsying squamous esophageal epithelium or gastric tissue instead of BE tissue (n = 8) and 3) and insufficient cells to perform FACS analysis (n = 2) [40]. This resulted in 31 BE patients and 18 age-matched controls that could be included in our study (for demographic data see Table 1). Controls were patients, who underwent upper endoscopy for upper gastrointestinal (GI) symptoms other than gastroesophageal reflux disease (GERD) symptoms and had no previous history of GERD and immune-associated disorders like celiac disease. Symptoms were evaluated by a standardised questionnaire, which needed to be negative for GERD symptoms. Controls were also not allowed to be known with immune-associated disorders. Of 12 included BE patients, biopsies were taken from BE and duodenum for FCS-analysis and immunohistochemical staining. Paired biopsies were taken from each section, with one biopsy being used for T-cell expansion cultures and one for immunohistochemical stainings. From 13 controls, biopsies were taken from duodenum (no endoscopic abnormalities) and used for FACS-analysis and immunohistochemical staining. Of 5 BE patients paired biopsies from BE tissues were used for validation of the *ex vivo* culture: one biopsy was taken for treatment with collagenase and one biopsy was used for *ex vivo* culture. Of 14 BE patients, biopsies were taken from BE and duodenum for mRNA isolation and QT-PCR. From 5 controls duodenal biopsies were used for mRNA isolation.

**Table 1:** Demographic data of patients with Barrett's esophagus (BE) and controls included in this study.

	BE patients	Controls
Number of patients	31	18
Mean age (±SD)	59±12	55±17
Gender (% males)	68%	19%
Presence of low grade dysplasia	19%	0
PPI use	97%	23%
Hiatal Hernia	81*%*	16%

The study was approved by Medical Ethical Committee of the University Medical Center Utrecht and written informed consent was obtained from all patients and controls.

### Immunohistochemistry

Biopsies were fixed in formalin and embedded in paraffin as described previously 7. In short, sections (4 µm) were deparaffinised and endogenous peroxidase was blocked by using 0.3% H_2_O_2_-blocking buffer (Sigma, St. Louise, MO, USA). For staining with anti-CD8, cytotoxic T-cell marker (clone c8/144B, DAKO, Glostrup, Denmark), anti-CD3 and pan-T-cell marker (polyclonal rabbit, DAKO), antigen retrieval was performed in 10 mM monocitric acid (pH = 6) at 100°C for 20 min. For staining with anti-CD4, a T-helper cell marker (clone 4B12, Monosan®, The Netherlands), antigen retrieval was performed in 10 mM Tris and 1 mM ethylenediamine tetraaceticacid (pH = 8) at 100°C for 20 min, while for staining with anti-MBP (Major Basic Protein) (clone BMK13, AbD Serotec, Dusseldorf, Germany), antigen retrieval was performed by treatment with pepsin in HCL-buffer (pH = 2) for 15 minutes at 37.5°C. The sections were stained with primary antibodies directed against cytotoxic T cells (CD8, 4 mg/L), T helper cells (CD4, 1∶100), total lymphocytes (CD3, 6 mg/L), and eosinophils (anti-MBP; 1∶4000, 50 ng/L) for 1 h at room temperature. Then, biotinylated horse anti-mouse IgG antibody (clone S0721, Vector, CA, USA, 3 mg/L) or biotinylated goat anti-rabbit antibody (clone R0919, Vector, CA, USA, 3 mg/L) was added, followed by incubation with streptavidin HRP (1∶1000, IM 0309, Beckman Coulter, Marseille, France), or by incubation with polyclonal anti-mouse, -rabbit, or -rat Powervision HRP (Immunologic, Duiven, The Netherlands). A brown color was developed using di-amino-benzidine substrate (Sigma) and bright DAB-substrate kit (BS04-110, Immunologic). The positive controls for anti-CD3, -CD4 and -CD8 (tonsil tissue) and anti-MBP stainings (nasal polyp tissue) were taken with each staining. Negative controls for the immunohistochemical stainings were obtained by omitting primary antibody from the staining protocol. Quantification of stained cells was performed by a computer assisted video microscopy system (Quantimet 570C, DXMRE microscope, PL fluotar 40× power objective lens (Leica, Heidelberg, Germany)) with custom made software which aids in the counting of cells and expresses the data as cells per mm^2^ per area measured. The number of cells per mm^2^ was determined in specialized intestinal epithelium (SIE) and lamina propria (LP) of BE and epithelium and LP of duodenum. When several biopsies from the same patient were present on a slide, the average of the values of all biopsies on each slide was calculated irrespective of the size of the biopsy.

### *Ex vivo* expansion of T-cells

Expansion of T-cells was performed according to a previously described method with small modifications [14]. In short, fresh biopsies were washed three times in IMDM medium (Lonza, Basel, Switzerland,) with 10 µ amphotericin (Fungizone®, Gibco™, Invitrogen, Camarillo, CA, USA). Cellfoam matrices ((9 mm×9 mm×1.5 mm; Cellsciences Pte Ltd, Singapore) were autoclaved, then incubated in 100 mg/ml rat tail collagen I (BD Biosciences, Bedford, MA) in phosphate-buffered saline (PBS) for 30 minutes at 37°C, followed by two rinses in PBS. Biopsies were placed on the matrix in a T-cell culture medium (IMDM, 14.2 M β-mercaptoethanol, penicillin, streptomycin, heat inactivated foetal calf serum (8%, Gibco™), 1 mM Hepes, and 0.29 mg/ml freshly added L-glutamine (Gibco™), with 10 units of IL-2 (BD Biosciences). Clark *et al*. previously showed that IL-2 alone increased proliferation by 5-fold while cells preserved their homing phenotype [14]. Cells were harvested after 1, 2 and 3 weeks of culturing for staining and analysis by flow cytometry (FACS) (FACScalibur, Becton&Dickinson, Mountain View, CA, USA).

### Staining of cell surface markers and analysis by FACS

The immunophenotyping of lymphocytes was performed on day 14 of culturing, as we determined in an earlier pilot study that the number of cells as measured by FACS was only sufficient after at least 2 weeks of culturing (results not shown). Cells (0.5×10^3^–10^5^) were washed with PBS supplemented with trisodium citrate (0.4% w/v, pH 7.4) and human pasteurised plasma solution (4 g/L; PBS2+) and subsequently incubated for 30 minutes on ice with directly labelled antibodies according to the instructions of the manufacturer. After washing with PBS2+, cells were resuspended in the same buffer and analysed by FACS.

For FACS stainings, the following antibodies were used: mAb CD3-FITC (clone sk7, 1∶20, BD Biosciences), CD3-PE (clone sk7, 1∶20, BD Biosciences), CD8-APC (clone SK1, 1∶100, BD Biosciences), CD8-PerCP(clone SK1, 1∶20, BD Biosciences), CD4-PerCP (clone L200, 1∶20, BD Pharmingen, San Diego, CA, USA), CD45RO-PE, memory T-cell marker (clone UCH1, 1∶25, BD Biosciences), CD45 RA-FITC, naïve T-cell marker (clone L48, 1∶25, BD Biosciences), CD103 (αE)-FITC, intraepithelial T-cell marker (Clone Ber-ACT8, 1∶20, BD Pharmingen), CD49d (α4)-PE (clone 9F10, 1∶20, eBioscience, San Diego, CA, USA), and anti-β7-PerCP (clone FIB27, BioLegend, San Diego, CA, USA). Positive populations for CD4, CD8, CD45RO, CD94 and NKg2a were identified by testing the specific antibodies together with the appropriate isotype control on PBMC's. Isotype controls were not taken along with the experiments of *ex vivo* cultured cells due to the small number of cells available.

### Intracellular granzyme B staining for FACS analysis

First, 2×10^3^–10^5^ cells were stained with the cell surface markers CD103 (αE)-FITC, Clone Ber-ACT8, BD Pharmingen) and CD8-PerCP (clone SK1, BD Biosciences). Then, cells were fixed in a fixation/permeabilization solution (eBioscience) for 10 minutes. After washing in a permeabilization solution (eBioscience), cells were incubated with mAb anti-granzyme B-PE (2 µg/ml, clone CLB-GB11, Sanquin, Amsterdam, The Netherlands) in the permeabilization solution (eBioscience) for 30 minutes. Cells were washed with PBS2+ and then FACS analysis was performed. Distinct populations were identified in lymphocytes from Barrett tissue and duodenal tissue. This enabled us to clearly distinguish populations with a high granzyme B expression level.

### Intracellular staining for IFN-γ and IL-4

For intracellular cytokine staining, 3-week cultures were chosen due to the requirement of a relatively high number of cells. Cells (2×10^3^–10^5^) in culture were stimulated for 6 hours with 3 µg/ml ionomycin (Invitrogen) and 20 ng/ml phorbol 12-myristate 13-acetate (ICN Biomedicals Inc, Aurora, OH, USA). One hour after the start of the stimulation brefeldin-A (Invitrogen) was added to stop the exocytosis of the cytokines. Cells were stained with the cell surface markers CD4-PerCp (1∶20, clone L200, BD Pharmingen) and CD8-APC (1∶100 clone SK1, BD Biosciences). Then, cells were fixed in 4% paraformaldehyde for 20 minutes on ice. Cells were permeabilised by 15-minutes incubation on ice in Perm/Wash Buffer (BD Biosciences). The next step was to incubate the cells 50 µl Perm/Wash buffer containing anti-IFN-γ-FITC (clone 25723.110, 1∶20) and IL-4 (clone 3010.211, 1∶20) (BD Biosciences) for 30 minutes on ice. The next step was to wash the cells twice in Perm/Wash buffer, after which the cells were taken up in PBS/0.1% sodium azide for FACS analysis.

### Treatment of biopsies with collagenase

Fresh biopsies were treated with collagenase III, 1 mg/ml (Worthington, Freehold, NJ, USA) for 1 hour in RPMI medium at 37°C and put through a cell strainer (BD Falcon™, BD Drive, NJ, USA). Then, the cells were washed with PBS2+, stained with the fluorescent labels CD103 (αE)-FITC, CD3-PE CD4-PerCP, CD8-APC and analysed with FACS.

### RT-PCR RNA analysis

RNA purified from esophageal biopsies was used for real-time PCR assays. RNA was isolated from from esophageal and duodenal biopsies using the RNeasy Mini Kit (Qiagen, Valencia, CA), according to the manufacturer's instructions. For cDNA synthesis, 1 µg total RNA was transcribed into cDNA with the cDNA transcription reagents (Bio-Rad, Hercules, CA, USA) using oligo(dT) and random primers according to manufacturer's instructions. Amplification and real time detection of PCR products with SYBR green was performed in a MyiQ Real-Time PCR detection system (Bio-Rad, Hercules, CA, USA) under the following conditions: 3 minutes at 95°C and 40 cycles of 30 sec at 95°C, 30 sec at 60°C and 30 sec at 72°C. The results represent relative quantity of mRNA levels normalised for the housekeeping gene GAPDH and plotted as fold change. Primers used were GAPDH F: 5′-agaaggctggggctcattt-′3, GAPDH R: 5′-gaggcattgctgatgatcttg-′3, (MAdCAM-1 F: 5′-acgcagggagaagtgatcccaaca-′3, MAdCAM-1 R: 5′-tttccagaggtgatacgtgggcaa-′3. GAPDH and MAdCAM-1 primers were purchased from Sigma Aldrich, St. Louis, USA. The expression level of a gene in a given sample was represented as 2^−ΔCT,^ where ΔCT = [CT_(experimental)_]−[CT_(housekeeping)_]. PCR assays were performed in duplicate.

### Statistical analyses

All continuous variables were statistically analysed with one-way analysis of variance non-parametric test, using Kruskal-Wallis test used to compare the three groups: BE tissue, duodenum of BE patients and duodenum of controls. A two-sided p-value <0.05 was considered to be statistically significant. All statistical analyses were conducted using GraphPad Prism 5 (La Jolla, CA, USA).
